# Higher intake of certain nutrients among older adults is associated with better cognitive function: an analysis of NHANES 2011–2014

**DOI:** 10.1186/s40795-023-00802-0

**Published:** 2023-12-05

**Authors:** Prasad P. Devarshi, Kelsey Gustafson, Ryan W. Grant, Susan Hazels Mitmesser

**Affiliations:** Science and Technology, Pharmavite, LLC, 8531 Fallbrook Ave, West Hills, CA 91304 USA

**Keywords:** Nutrient gaps, Nutrient adequacy, Dietary reference intakes, Cognition, Depression, Older adults

## Abstract

**Background:**

An increasing number of adults are over the age of 65, and there is concern about the increasing prevalence of age-associated cognitive decline and poor mental health status in older adults in the United States. Several nutrients are known to have important biological roles in brain health and neurological function, but many individuals fall short of recommended intake levels. The objective of this study was to examine the association between nutrient intake and cognitive function. We also explored whether nutrient intake was associated with depression.

**Methods:**

This cross-sectional study was based on data from the National Health and Nutrition Examination Survey (NHANES) 2011–2014 and included participants ≥ 60 years of age who had reliable day 1 dietary recall data and either valid cognitive function data (*n* = 2713) or valid depression score data (*n* = 2943). The sample was stratified by gender, and cognitive functioning test (CFT) composite z-scores were analyzed by quartiles. Depression status was assessed using the Patient Health Questionnaire (PHQ-9).

**Results:**

Higher intake and adequacy of a number of different nutrients from food were associated with higher cognitive function in both males and females. Nutrients that showed the most consistent associations with cognitive function across intake and adequacy analyses for food in both males and females were vitamin A, vitamin E, thiamin, riboflavin, vitamin B6, folate, magnesium, potassium, zinc, vitamin K, and lutein and zeaxanthin (*p* < 0.05 for all). These associations were positive with increasing intake and adequacy being associated with higher CFT composite z-scores. Analysis of nutrient intake and depression yielded results that differed by gender. In females, the nutrients that showed consistent inverse associations with depression scores across both intake and adequacy analyses for food were vitamin A, vitamin C, magnesium, vitamin K, potassium, and dietary fiber (*p* < 0.05 for all). In males, no significant associations between nutrient intake from food and depression scores were observed.

**Conclusions:**

Our findings suggest that older adults with sufficient intakes of certain essential nutrients have higher cognitive function. Future studies are needed to confirm whether a well-balanced diet and/or dietary supplements which emphasize these nutrients are effective for prevention of age-related declines in cognitive function and mood.

**Supplementary Information:**

The online version contains supplementary material available at 10.1186/s40795-023-00802-0.

## Introduction

Cognition encompasses a wide array of mental processes including attention, memory, executive function, language, and visuospatial abilities. Cognition is critical for individuals to be able to maintain functional independence, communicate effectively with others, and maintain quality of life. As part of the normal aging process, cognitive abilities decline, specifically those related to cognitive tasks that require one to quickly process or transform information to make a decision [1]. Age-associated declines in cognition are especially of concern due to the increasing number of adults over the age of 65 in the United States [1]. In 2021, there were 56 million adults aged 65 and older comprising 17% of the US population [2]. By 2050, this number is expected to increase to 80 million [3]. Approximately 12% of adults aged 65 and older report confusion or memory problems that have been happening more often or getting worse within the last year [4]. Additionally, 22% of US adults have mild cognitive impairment [5]. Cognition has also been linked to mood as symptoms of depression and anxiety appear to be more prevalent in people with mild cognitive impairment compared with people with normal cognitive function [6]. It is estimated that the prevalence of major depression is 1–5% in older adults in the US, but rates can be much higher in those who are hospitalized or require home healthcare [7]. There is emerging evidence that lifestyle factors such as a healthy diet and/or specific nutrients may slow age-related cognitive decline and may help delay the onset of cognitive symptoms, thus helping to preserve quality of life [8].

Previous studies have observed that better diet quality is associated with improved cognitive performance in older adults, while conversely nutritional deficiencies can worsen cognitive deterioration [9–11]. Several nutrients are known to have important biological roles in brain health and neurological function. Vitamin B12 is necessary for the synthesis and maintenance of myelin, the protective coating around nerve fibers [12]. Magnesium has an essential role in nerve transmission and neuromuscular conduction [13]. Retinoic acid, the active metabolite of Vitamin A, modulates neurogenesis, neuronal survival and synaptic plasticity [14]. Folate and choline are critical for fetal brain development [15, 16], and a recent study found that choline supplementation may improve memory performance in older adults with age-associated memory impairment [17]. Additionally, antioxidants such as vitamin E and lutein may protect the brain from damage by free radicals. Despite the biological importance of these nutrients, many individuals fall short of recommended intake levels. The 2020–2025 Dietary Guidelines for Americans identified vitamin D, calcium, dietary fiber, and potassium as food components of public health concern because of underconsumption. Additionally, vitamins A, C, E, and K, magnesium, and choline were identified as nutrients that are underconsumed, but which lack sufficient adverse health outcome data to warrant classification as a public health concern [18].

The objective of this study was to examine the association between nutrient intake and cognitive function. We also explored whether nutrient intake was associated with depression.

## Materials and methods

### Study population

This cross-sectional study is based on data from the National Health and Nutrition Examination Survey (NHANES), which provides nationally representative descriptive health and nutrition statistics for the United States [19]. NHANES data has been collected continuously since 1999, and data are released publicly in 2-year cycles. The NHANES study protocol was approved by the National Center for Health Statistics Research Ethics Review Board, and all participants provided written informed consent [19]. NHANES includes a household interview conducted at the respondent’s home and a health examination conducted in specially-designed and equipped mobile examination centers [19]. As part of the household interview, all participants were eligible to complete the Dietary Supplement and Prescription Medication Section (DSQ) of the Sample Person Questionnaire with a trained interviewer.

As part of the NHANES data collection, cognitive functioning testing was only conducted among populations ≥ 60 years of age but not performed in all NHANES cycles. The most recent NHANES cycles that included the cognitive functioning component are the 2011–12 and 2013–14 cycles. Hence, in the current assessment, the study population consisted of NHANES participants age ≥ 60 years who participated in the 2011–12 and 2013–14 cycles of NHANES (NHANES 2011–14).

**Fig. 1:**
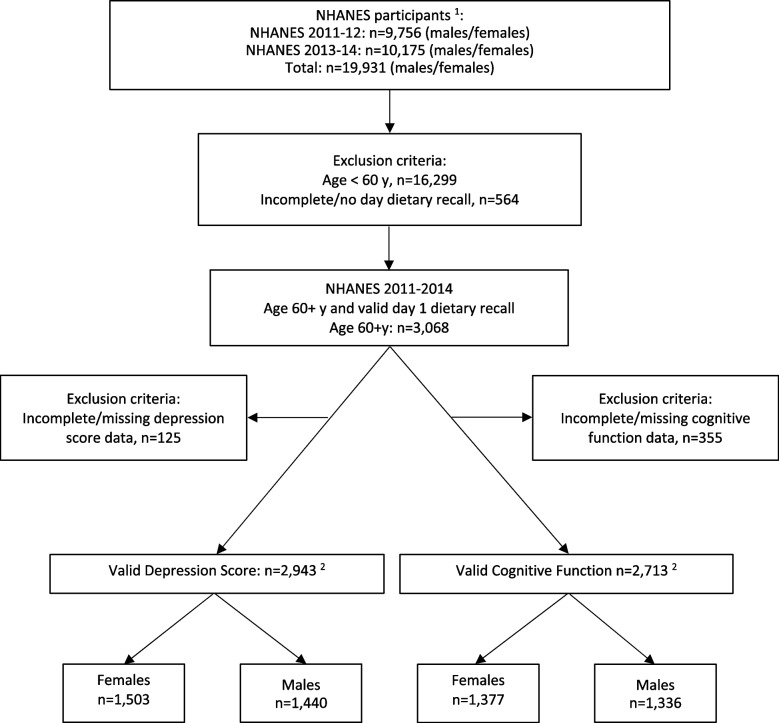
Eligible sample population. ^1^All participants were eligible to complete the Dietary Supplement and Prescription Medication Section (DSQ). ^2^The population size is for the total population with a valid depression score or cognitive function data. Analyses using model 2 had a smaller sample size due to missing covariates

Only individuals with a reliable Day 1 dietary recall (determined by the Centers for Disease Control National Center for Health Statistics as reliable and meeting the minimum criteria set) were considered in the analysis (Fig. 1). Additionally, only participants with a valid cognitive function questionnaire or depression screener were included in the cognitive function and depression status analyses, respectively. Participants who were < 60 years of age or had incomplete or missing dietary recall data were excluded.

### Nutrient intake

NHANES survey personnel collected data on nutrient intake from food and supplements during the household interview. Estimated nutrient intakes from food were based on two 24-h dietary recalls, and estimated nutrient intake from supplements were based on the 30-day supplement intake data and 24-h dietary recall [20]. The second 24-h dietary recall interview was administered by telephone three to ten days after the first dietary interview. Total daily nutrient intake data for Day 1 and 2 from NHANES 2011–14 and total nutrient intake from the 30-day supplement use for NHANES 2011–14 were compiled. Intake from supplements for the nutrients that have not been processed by NHANES [vitamins A and E, and the two polyunsaturated fatty acids PUFA 20:5 (eicosapentaenoic) and PUFA 22:6 (docosahexaenoic)] were derived using the individual supplement intake and composition data compiled by NHANES. National Academy of Science, Engineering, and Medicine (NASEM) dietary reference intake (DRI) values were used to determine nutrient adequacy of the nutrients of interest. The prevalence of inadequate intake was derived using the cut-point method based on usual intake relative to the EAR or AI for nutrients with a DRI [21].

### Cognitive function

Cognitive functioning testing was conducted by a trained interviewer at the beginning of the face-to-face private interview and in the mobile examination center. The components of the cognitive testing included: 1) word learning and recall modules from the Consortium to Establish a Registry for Alzheimer’s Disease (CERAD); 2) the Animal Fluency test; and 3) the Digit Symbol Substitution Test (DSST) [22]. Cognitive testing was conducted in the participant’s preferred language (English, Spanish, Korean, Vietnamese, Chinese).

Subjects were classified into quartiles based on each of the following cognitive function tests: (i) the sum of all three CERAD Word List Learning Tests (immediate recall/learning), (ii) CERAD Word List Recall Test (delayed recall), (iii) Animal Fluency Test and (iv) DSST. The cognitive function test (CFT) composite score was derived by summing the z-scores on each of the four tests.

### Depression

The Patient Health Questionnaire (PHQ-9) was scored, and subjects were classified by depression status [23–25]. For each subject, a total score based on answers to each of the nine individual items (Have little interest in doing things; Feeling down, depressed, or hopeless; Trouble sleeping or sleeping too much; Feeling tired or having little energy; Poor appetite or overeating; Feeling bad about yourself; Trouble concentrating on things; Moving or speaking slowly or too fast; Thought you would be better off dead) included in the PHQ-9 was derived. Each of the 9 items can be scored from 0 (“not at all”) to 3 (“nearly every day”) with a total score range of 0–27 [23]. Subjects with missing answers on one or more of the nine questionnaire items were excluded. The total scores were categorized as follows: minimal depression (0–4), mild depression (5–9), moderate depression (10–14), moderately severe depression (15–19), and severe depression (20–27). These were then grouped into two categories, minimal/mild depression (0–9) and moderate/moderately severe/severe depression (10–27) based on the cut-off score of 10 for clinically significant depressive symptoms on the PHQ-9.

### Covariates

The factors considered as potential confounders in multivariable modeling were age, race/ethnicity, household income, education, marital status, body mass index (BMI), cigarette smoking status, alcohol use status, antidepressant use status, physical activity level, and presence of comorbidities (coronary heart disease, stroke, high blood pressure, diabetes, cancer, and impaired renal function).

Demographic characteristics were categorized as follows: age (60 to < 70 y, 70 to < 80 y, and ≥ 80 y; gender (male and female); race and Hispanic origin (non-Hispanic White, non-Hispanic Black, non-Hispanic Asian, Hispanic, and other); household poverty to income ratio (PIR) (≤ 1.35 of PIR, > 1.35 to < 1.85 of PIR, ≥ 1.85 of PIR); education (less than high school, high school graduate or general equivalency diploma, some college or associates degrees, and bachelor’s degree or above); and marital status (never been married, divorced/widowed, currently married). Lifestyle characteristics were categorized as follows: smoking status (never smoker, past smoker, current smoker); number of alcoholic drinks per day (0, 1, > 1); and physical activity (< 10 min moderate physical activity equivalent/week, 10 – 150 min/week, ≥ 150 min/week).

Information on medical history and health conditions was collected as part of a series of questionnaires collected in the mobile examination center. In addition, body measures and biological samples were also collected as part of the examination. Antidepressant use over the previous 30-day period was based on response to The Dietary Supplement and Prescription Medication section of the Sample Person Questionnaire. Comorbidities and health characteristics were categorized as follows: BMI (underweight, normal weight, overweight, obese); ever told you had coronary heart disease (yes, no); ever told you had a stroke (yes, no); ever told you had cancer or malignancy (yes, no); ever told you had high blood pressure (yes, no); ever told you had diabetes (yes, no); antidepressant use (yes, no); and impaired renal function (glomerular filtration rate (GFR) ≥ 60 mL/min per 1.73 m^2^ and/or albumin-to-creatine ratio (ACR) < 30 mg/g, GFR < 60 mL/min per 1.73 m^2^ & ACR ≥ 30 mg/g).

### Statistical analysis

Statistical analyses were performed with SAS software (Version 9.4, SAS Institute Inc., Cary, NC, USA) and STATA (Version 12.1, StataCorp LP, College Station, TX, USA). All analyses used statistical weights adjusted for survey design and were stratified by gender.

Usual intake of nutrients from dietary sources (food and supplements) was estimated using the National Cancer Institute (NCI) method and the SAS macros developed by NCI for modeling of a single dietary component [26, 27]. The “shrink then add” approach was used to estimate the usual intake from dietary and supplemental sources combined [28]. The resulting combined intakes were processed to obtain the final distributions of usual intakes from dietary and supplemental sources combined. The following covariates were used in the analysis: day of the week (weekday/weekend), sequence of the dietary recall (day 1 or 2), whether the participant reported use of any dietary supplement in the past 30 days, and age (60–69 y, 70–79 y, 80 + y). Balanced repeated replicate weights (Fay adjustment factor = 0.3) based on day 1 dietary recall statistical weights were used for estimation of standard errors (SE) [29]. The prevalence of inadequate intake was derived using the cut-point method based on usual intake relative to the estimated average requirement (EAR) or adequate intake (AI) for nutrients with a DRI. Calcium is the only nutrient included in this analysis that has a different EAR by age among males (800 mg for males 51- 70 y versus 1000 mg for males > 70 y). Given the interest in determining nutrient adequacy for the study population (and not by DRI age group), the minimum EAR (i.e. 800 mg) was used to conservatively estimate nutrient adequacy of calcium among males.

Linear regression models were used to evaluate the association between CFT composite scores and nutrient intake levels. Participants were classified in the four cognitive performance quartile groups for each of the four cognitive function tests listed above and the composite score, and mean usual intake estimates and prevalence of inadequate intake across the categories were derived using the NCI macros. Two models were run for each of the nutrients of interest. Model 1 included the same covariates included in the usual intake (UI) assessment of the total population (i.e. indicators day of the week, sequence of the dietary recall, dietary supplement use in the past 30 days, and age) in addition to an indicator variable for cognitive function. Model 2 included the same covariates as Model 1 with additional indicator variables for education, race/ethnicity, marital status, PIR, comorbidities (coronary heart disease, stroke, hypertension, diabetes, cancer), renal function (GFR and albuminuria), BMI, smoking status, physical activity, and alcohol intake. Means of usual nutrient intake distributions and percent below the EAR or above the AI of the three lowest quartile categories were compared to the mean intake for the higher quartile category using a z-test. Analyses using model 1 are not presented here.

Similar analyses were conducted to evaluate the association between total depression score and nutrient intakes. Participants were classified into the two depression groups (scores < 10 vs ≥ 10 as described above) and mean usual intake estimates and prevalence of inadequate intake across the categories were derived using the NCI macros. Means of usual nutrient intake distributions and percent below the EAR or above the AI for the two depression groups were compared by computing a Z-statistic.

## Results

### Population characteristics

Figure 1 shows how the eligible sample population for this study was determined from all NHANES participants. In total, 2713 individuals were included in cognitive function analyses and 2943 individuals were included in depression analyses. Table 1 summarizes demographic and lifestyle characteristics of the study population divided by gender. The majority of the study population was non-Hispanic white (77.3%), with a household income greater than 1.85 times the poverty to income ratio (69.3%). Most of the study population had more than a high school education level (59.6%) and were married (61.8%). Overall, 12.4% of men and 23.4% of women had used an antidepressant within the previous 30 days. Information on comorbidities that were included as covariates can be found in Supplementary Table S1.

**Table 1:** Summary characteristics of the study population

**Demographic Characteristic**	**Total**		**Male**		**Female**	
	**n**	**Mean ± SE**	**n**	**Mean ± SE**	**n**	**Mean ± SE**
Age (y)	3068	69.5 ± 0.2	1509	69.2 ± 0.3	1559	69.7 ± 0.2
Race/ethnicity (%)	3068		1509		1559	
Mexican American	273	3.8 ± 0.9	146	4.2 ± 1.0	127	3.5 ± 0.9
Other Hispanic	301	3.9 ± 0.7	138	3.5 ± 0.6	163	4.3 ± 0.9
Non-Hispanic white	1463	77.3 ± 2.0	699	78.1 ± 2.0	764	76.6 ± 2.0
Non-Hispanic black	740	9.1 ± 1.3	380	8.4 ± 1.2	360	9.6 ± 1.4
Other race (including multiracial)	291	5.9 ± 0.7	146	5.8 ± 0.7	145	6.0 ± 0.9
Household income (%)	2822		1388		1434	
≤ 1.35 of PIR	939	20.6 ± 1.5	428	17.2 ± 1.3	511	23.4 ± 1.9
> 1.35 to < 1.85 of PIR	328	10.2 ± 1.0	157	8.0 ± 1.1	171	12.0 ± 1.1
≥ 1.85 of PIR	1555	69.3 ± 2.0	803	74.8 ± 1.7	752	64.5 ± 2.5
Education (%)	3064		1506		1558	
< High school	859	18.1 ± 1.5	429	17.8 ± 1.7	430	18.4 ± 1.8
High school	708	22.3 ± 1.3	340	20.2 ± 1.8	368	24.1 ± 1.6
> High school	1497	59.6 ± 1.7	737	61.9 ± 1.8	760	57.6 ± 2.0
Marital status (%)	3065		1508		1557	
Never been married	266	7.0 ± 0.7	141	8.3 ± 1.0	125	5.9 ± 0.6
Divorced/Widowed	1139	31.3 ± 0.9	371	17.8 ± 1.2	768	42.6 ± 1.3
Currently married	1660	61.8 ± 1.3	996	73.9 ± 1.6	664	51.5 ± 1.5
BMI (%)	3016		1481		1535	
Underweight	47	1.4 ± 0.3	19	1.2 ± 0.4	28	1.6 ± 0.4
Normal	777	25.5 ± 1.2	378	22.7 ± 1.5	399	27.9 ± 1.6
Overweight	1055	35.8 ± 1.2	602	40.9 ± 2.1	453	31.5 ± 1.3
Obese	1137	37.3 ± 1.4	482	35.2 ± 2.1	655	39.1 ± 1.8
Smoking status (%)	3065		1508		1557	
Current smoker	383	10.8 ± 0.6	230	12.6 ± 0.9	153	9.2 ± 0.9
Past smoker	1159	39.3 ± 1.4	728	48.6 ± 1.7	431	31.4 ± 1.9
Never smoked	1523	50.0 ± 1.5	550	38.8 ± 1.9	973	59.4 ± 1.8
Number of alcoholic drinks per day (%)	2989		1467		1522	
0	1372	38.4 ± 1.8	578	32.7 ± 1.7	794	43.2 ± 2.3
1	877	35.4 ± 1.4	375	29.2 ± 1.4	502	40.6 ± 2.2
> 1	740	26.3 ± 1.4	514	38.1 ± 1.7	226	16.2 ± 1.4
Physical activity (%)	3065		1507		1558	
< 10 min per week	1488	47.2 ± 1.7	695	44.2 ± 1.8	793	49.7 ± 2.0
10 to < 150 min per week	538	16.2 ± 1.0	249	15.1 ± 1.5	289	17.2 ± 1.0
≥ 150 min per week	1039	36.6 ± 1.3	563	40.7 ± 1.4	476	33.1 ± 1.8
Antidepressant use (%)	3066		1509		1557	
No	2597	81.6 ± 0.9	1354	87.6 ± 1.2	1243	76.6 ± 1.3
Yes	469	18.4 ± 0.9	155	12.4 ± 1.2	314	23.4 ± 1.3

*BMI* body mass index, *PIR* poverty-to-income ratio

### Associations between nutrient intake and cognitive function in females

Usual nutrient intake from food alone and food + supplements and nutrient intake adequacy, expressed as percent below the EAR or percent above the AI for nutrients without an EAR, is provided in Supplementary Tables S2 and S3 for males and females, respectively. In females, higher intakes of vitamin A, vitamin E, thiamin, riboflavin, niacin, vitamin B6, folate, vitamin B12, iron, magnesium, phosphorous, zinc, copper, selenium, vitamin K, choline, potassium, dietary fiber, and lutein + zeaxanthin were associated with higher CFT composite z-scores (*p* < 0.05 for all, Table 2). Intake of vitamin A, vitamin E, riboflavin, magnesium, phosphorus, copper, vitamin K, potassium, and lutein + zeaxanthin from food alone was significantly lower among the first (Q1) and second (Q2) CFT composite z-score quartiles compared with the highest quartile (Q4) (*p* < 0.05 for all). Intake of thiamin, niacin, vitamin B6, folate, vitamin B12, iron, zinc, selenium, total choline, and dietary fiber from food alone was significantly lower among the first (Q1) CFT composite z-score quartile compared with the highest quartile (Q4) (*p* < 0.05 for all).

**Table 2:** Nutrient intake from food alone by cognitive function status among females 60 ^+^ years of age^†^

		**Total CFT Composite Z-Score**					
		**Z-score Q1**		**Z-score Q2**		**Z-score Q3**	**Z-score Q4**
		***n* = 352**		***n* = 313**		***n* = 279**	***n* = 204**
**Nutrient**	**EAR**	**Mean ± SE**		**Mean ± SE**		**Mean ± SE**	**Mean ± SE**
Vitamin A, RAE (μg)	500	568 ± 21	^*^	567 ± 25	^*^	667 ± 30	666 ± 27
Vitamin E as alpha-tocopherol (mg)	12	6.4 ± 0.2	^*^	7.1 ± 0.3	^*^	8.7 ± 0.4	9.1 ± 0.5
Vitamin D (D2 + D3) (μg)	10	4.2 ± 0.3		4.1 ± 0.2		4.2 ± 0.3	4.6 ± 0.4
Vitamin C (mg)	60	73 ± 3		74 ± 6		86 ± 5	83 ± 4
Thiamin (Vitamin B1) (mg)	0.9	1.21 ± 0.03	^*^	1.31 ± 0.04		1.35 ± 0.05	1.43 ± 0.05
Riboflavin (Vitamin B2) (mg)	0.9	1.67 ± 0.04	^*^	1.76 ± 0.05	^*^	1.84 ± 0.06	1.98 ± 0.07
Niacin (mg)	11	17.4 ± 0.5	^*^	19 ± 0.5		20 ± 0.7	21 ± 0.7
Vitamin B6 (mg)	1.3	1.51 ± 0.05	^*^	1.6 ± 0.06		1.72 ± 0.07	1.76 ± 0.06
Folate, DFE (μg)	320	419 ± 12	^*^	456 ± 19		455 ± 17	479 ± 17
Vitamin B12 (μg)	2	3.7 ± 0.1	^*^	3.8 ± 0.2		4.1 ± 0.2	4.4 ± 0.4
Calcium (mg)	1000	780 ± 26		808 ± 29		809 ± 29	884 ± 41
Iron (mg)	5	11.9 ± 0.3	^*^	12.1 ± 0.4		12.4 ± 0.5	13 ± 0.4
Magnesium (mg)	265	235 ± 7	^*^	249 ± 7	^*^	275 ± 8	291 ± 11
Phosphorus (mg)	580	1019 ± 28	^*^	1097 ± 31	^*^	1140 ± 30	1230 ± 46
Zinc (mg)	6.8	8.1 ± 0.3	^*^	8.7 ± 0.3		9 ± 0.3	9.9 ± 0.4
Copper (mg)	0.7	1 ± 0.03	^*^	1.1 ± 0.04	^*^	1.2 ± 0.04	1.2 ± 0.05
Selenium (μg)	45	79 ± 2	^*^	92 ± 3		91 ± 3	98 ± 3
	**AI**						
Vitamin K (μg)	90	95 ± 5	^*^	101 ± 6	^*^	147 ± 9	147 ± 9
Total choline (mg)	425	237 ± 8	^*^	266 ± 9		279 ± 11	293 ± 12
Potassium (mg)	2600	2171 ± 60	^*^	2279 ± 65	^*^	2485 ± 69	2578 ± 89
Dietary fiber (g)	21	14.5 ± 0.6	^*^	15 ± 0.5		16.7 ± 0.6	16.8 ± 0.7
Lutein + zeaxanthin (μg)	NA	1386 ± 111	^*^	1502 ± 112	^*^	2252 ± 229	2056 ± 179
PFA 20:5 (Eicosapentaenoic) (g)	NA	0.02 ± 0.002		0.02 ± 0.002		0.02 ± 0.004	0.02 ± 0.004
PFA 22:6 (Docosahexaenoic) (g)	NA	0.05 ± 0.008		0.08 ± 0.009		0.09 ± 0.013	0.09 ± 0.016

^†^Model 2: covariates for day of the week (weekday/weekend), sequence of the dietary recall (day 1 or 2), whether the participant reported use of any dietary supplement in the past 30 days, and age (60–69 y, 70–79 y, 80 + y), education, race/ethnicity, marital status, PIR, comorbidities (CHD, stroke, hypertension, diabetes, cancer), kidney function (GFR and albuminuria), BMI, smoking status, physical activity, and alcohol intake

^*^Statistically significant difference (*p* < 0.05) compared to Q4

### Associations between nutrient intake and cognitive function in males

In males, higher intakes of vitamin A, vitamin E, thiamin, riboflavin, niacin, vitamin B6, folate, vitamin B12, calcium, iron, magnesium, phosphorous, zinc, copper, selenium, vitamin K, choline, potassium, dietary fiber, and lutein + zeaxanthin from food alone were associated with higher CFT composite z-scores (*p* < 0.05 for all, Table 3). Intake of vitamin E, thiamin, riboflavin, magnesium, phosphorus, copper, vitamin K, total choline, potassium, and dietary fiber from food alone was significantly lower among the first (Q1) and second (Q2) CFT composite z-score quartiles compared with the highest quartile (Q4) (*p* < 0.05 for all). Intake of vitamin A, niacin, vitamin B6, folate, vitamin B12, calcium, iron, zinc, selenium, and lutein + zeaxanthin from food alone was significantly lower among the first (Q1) CFT composite z-score quartile compared to the highest quartile (Q4) (*p* < 0.05 for all).

**Table 3:** Nutrient intake from food alone by cognitive function status among males 60 ^+^ years of age^†^

		**Total CFT Composite Z-Score**					
		**Z-score Q1**		**Z-score Q2**		**Z-score Q3**	**Z-score Q4**
		***n* = 395**		***n* = 301**		***n* = 231**	***n* = 198**
**Nutrient**	**EAR**	**Mean ± SE**		**Mean ± SE**		**Mean ± SE**	**Mean ± SE**
Vitamin A, RAE (μg)	625	670 ± 43	^*^	729 ± 60		861 ± 100	875 ± 56
Vitamin E as alpha-tocopherol (mg)	12	8 ± 0.3	^*^	9 ± 0.5	^*^	11 ± 0.4	12 ± 0.6
Vitamin D (D2 + D3) (μg)	10	4.9 ± 0.3		5.3 ± 0.4		5.6 ± 0.4	5.8 ± 0.4
Vitamin C (mg)	75	86 ± 5		89 ± 8		85 ± 7	103 ± 8
Thiamin (Vitamin B1) (mg)	1	1.53 ± 0.06	^*^	1.73 ± 0.07	^*^	1.88 ± 0.07	1.95 ± 0.06
Riboflavin (Vitamin B2) (mg)	1.1	2.07 ± 0.07	^*^	2.35 ± 0.1	^*^	2.56 ± 0.13	2.71 ± 0.11
Niacin (mg)	12	24 ± 1	^*^	27 ± 1		29 ± 1	29 ± 1
Vitamin B6 (mg)	1.4	1.97 ± 0.09	^*^	2.27 ± 0.08		2.4 ± 0.09	2.48 ± 0.1
Folate, DFE (μg)	320	496 ± 22	^*^	572 ± 26		636 ± 28	651 ± 28
Vitamin B12 (μg)	2	4.9 ± 0.3	^*^	6.0 ± 0.4		6.3 ± 0.4	6.4 ± 0.5
Calcium (mg)	800^a^	880 ± 24	^*^	937 ± 53		1042 ± 44	1092 ± 46
Iron (mg)	6	14.9 ± 0.7	^*^	16.3 ± 0.7		18.1 ± 0.7	18 ± 0.6
Magnesium (mg)	350	286 ± 8	^*^	316 ± 9	^*^	349 ± 12	378 ± 16
Phosphorus (mg)	580	1281 ± 34	^*^	1428 ± 50	^*^	1525 ± 45	1632 ± 47
Zinc (mg)	9.4	10.6 ± 0.4	^*^	11.8 ± 0.4		13 ± 0.4	13.1 ± 0.47
Copper (mg)	0.7	1.2 ± 0.04	^*^	1.3 ± 0.05	^*^	1.5 ± 0.06	1.6 ± 0.07
Selenium (μg)	45	106 ± 3	^*^	120 ± 4		126 ± 4	127 ± 3
	**AI**						
Vitamin K (μg)	120	100 ± 5	^*^	113 ± 6	^*^	123 ± 12	157 ± 14
Total choline (mg)	550	329 ± 11	^*^	362 ± 10	^*^	398 ± 16	401 ± 10
Potassium (mg)	3400	2680 ± 62	^*^	2944 ± 88	^*^	3219 ± 150	3390 ± 101
Dietary fiber (g)	30	17.2 ± 0.7	^*^	18.2 ± 0.8	^*^	19.8 ± 1.0	22.4 ± 1.1
Lutein + zeaxanthin (μg)	NA	1337 ± 120	^*^	1499 ± 143		1572 ± 166	2061 ± 248
PFA 20:5 (Eicosapentaenoic) (g)	NA	0.02 ± 0.003		0.03 ± 0.003		0.03 ± 0.005	0.03 ± 0.003
PFA 22:6 (Docosahexaenoic) (g)	NA	0.08 ± 0.011		0.10 ± 0.014		0.10 ± 0.019	0.11 ± 0.016

^†^Model 2: covariates for day of the week (weekday/weekend), sequence of the dietary recall (day 1 or 2), whether the participant reported use of any dietary supplement in the past 30 days, and age (60–69 y, 70–79 y, 80 + y), education, race/ethnicity, marital status, PIR, comorbidities (CHD, stroke, hypertension, diabetes, cancer), kidney function (GFR and albuminuria), BMI, smoking status, physical activity, and alcohol intake

^*^Statistically significant difference (*p* < 0.05) compared to Q4

^a^EAR for males 51—70 y is 800 mg and the EAR for males 71 + y is 1000 mg. An EAR of 800 mg was selected to conservatively estimate the percent below the EAR

### Associations between nutrient adequacy and cognitive function in females

The first quartile of CFT composite z-scores had a greater percentage of females with nutrient shortfalls (Table 4). The percentage of individuals consuming below the EAR for vitamin A, vitamin E, magnesium, and copper from food alone was significantly lower among females in the highest (Q4) CFT composite z-score quartile compared with the first (Q1) and second (Q2) quartiles (*p* < 0.05 for all). The percentage of individuals consuming below the EAR for thiamin, riboflavin, niacin, B6, folate, phosphorus, zinc, and selenium from food alone was significantly lower among females in the highest (Q4) CFT composite z-score quartile compared with the lowest quartile (Q1) (*p* < 0.05 for all). The percentage of individuals consuming above the AI for vitamin K and potassium from food alone was significantly higher among females in the highest (Q4) CFT composite z-score quartile compared with the first (Q1) and second (Q2) quartiles (*p* < 0.05 for all).

**Table 4:** Nutrient adequacy from food alone by cognitive function status among females 60 ^+^ years of age^†^

		**Total CFT Composite Z-Score**					
		**Z-score Q1**		**Z-score Q2**		**Z-score Q3**	**Z-score Q4**
		***n* = 352**		***n* = 313**		***n* = 279**	***n* = 204**
		**% < EAR**^‡^					
**Nutrient**	**EAR**	**% ± SE**		**% ± SE**		**% ± SE**	**% ± SE**
Vitamin A, RAE (μg)	500	44 ± 3.5	^*^	44 ± 4.1	^*^	29 ± 4.4	28 ± 4.0
Vitamin E as alpha-tocopherol (mg)	12	97 ± 1.2	^*^	94 ± 1.4	^*^	85 ± 3.7	82 ± 4.4
Vitamin D (D2 + D3) (μg)	10	97 ± 1.3		97 ± 0.9		97 ± 1.3	96 ± 1.6
Vitamin C (mg)	60	46 ± 2.7		44 ± 4.5		34 ± 4.1	36 ± 3.9
Thiamin (Vitamin B1) (mg)	0.9	19 ± 2.1	^*^	12 ± 2.6		10 ± 2.6	6 ± 2.5
Riboflavin (Vitamin B2) (mg)	0.9	6 ± 0.9	^*^	4 ± 0.9		< 3	< 3
Niacin (mg)	11	6 ± 1.7	^*^	< 3		< 3	< 3
Vitamin B6 (mg)	1.3	37 ± 4.3	^*^	29 ± 3.5		22 ± 4.1	19 ± 3.7
Folate, DFE (μg)	320	28 ± 2.7	^*^	20 ± 3.4		21 ± 3.9	16 ± 3.7
Vitamin B12 (μg)	2	10 ± 2.7		8 ± 1.8		6 ± 2.0	4 ± 1.4
Calcium (mg)	1000	81 ± 2.9		78 ± 3.8		78 ± 3.4	69 ± 5.4
Iron (mg)	5	< 3		< 3		< 3	< 3
Magnesium (mg)	265	70 ± 3.6	^*^	62 ± 3.9	^*^	49 ± 4.0	40 ± 5.1
Phosphorus (mg)	580	4 ± 1.2	^*^	< 3		< 3	< 3
Zinc (mg)	6.8	33 ± 4.5	^*^	25 ± 4.2		21 ± 3.8	13 ± 4.4
Copper (mg)	0.7	19 ± 2.6	^*^	13 ± 3.1	^*^	7 ± 1.7	4 ± 1.6
Selenium (μg)	45	3 ± 1.2	^*^	< 3		< 3	< 3
	**AI**	**% > AI**^‡^					
Vitamin K (μg)	90	44 ± 3.6	^*^	49 ± 4.6	^*^	75 ± 4.5	77 ± 4.7
Total choline (mg)	425	< 3		< 3		< 3	4 ± 1.8
Potassium (mg)	2600	23 ± 3.4	^*^	28 ± 3.6	^*^	41 ± 4.1	46 ± 5.4
Dietary fiber (g)	21	13 ± 3.1		14 ± 2.1		22 ± 3.1	22 ± 3.4

^†^Model 2: covariates for day of the week (weekday/weekend), sequence of the dietary recall (day 1 or 2), whether the participant reported use of any dietary supplement in the past 30 days, and age (60–69 y, 70–79 y, 80 + y), education, race/ethnicity, marital status, PIR, comorbidities (CHD, stroke, hypertension, diabetes, cancer), kidney function (GFR and albuminuria), BMI, smoking status, physical activity, and alcohol intake

^‡^Estimated values of % < EAR or % > AI less than 3% or greater than 97% are expressed “ < 3” and “ > 97”, respectively, and the SE’s are not displayed, in accordance with the USDA’s practice in reporting nutrient adequacy. Statistical comparisons were conducted using the estimated values < 3% or > 97%

^*^Statistically significant difference (*p* < 0.05) compared to Q4

### Associations between nutrient adequacy and cognitive function in males

The first quartile of CFT composite z-scores had a greater percentage of males with nutrient shortfalls (Table 5). The percentage of individuals consuming below the EAR for vitamin A, E, riboflavin, and magnesium from food alone was significantly lower among males in the highest (Q4) CFT composite z-score quartile compared with the first (Q1) and second (Q2) quartiles (*p* < 0.05 for all). The percentage of individuals consuming below the EAR for thiamin, B6, folate, vitamin B12, calcium, and zinc from food alone was significantly lower among males in the highest (Q4) CFT composite z-score quartile compared with the lowest quartile (Q1) (*p* < 0.05 for all). The percentage of individuals consuming above the AI for vitamin K, potassium, and dietary fiber from food alone was significantly higher among males in the highest (Q4) CFT composite z-score quartile compared with the second (Q2) quartile (*p* < 0.05 for all).

**Table 5:** Nutrient adequacy from food alone by cognitive function status among males 60 ^+^ years of age^†^

		**Total CFT Composite Z-Score**					
		**Z-score Q1**		**Z-score Q2**		**Z-score Q3**	**Z-score Q4**
		***n* = 395**		***n* = 301**		***n* = 231**	***n* = 198**
		**% < EAR**^‡^					
**Nutrient**	**EAR**	**% ± SE**		**% ± SE**		**% ± SE**	**% ± SE**
Vitamin A, RAE (μg)	625	54 ± 4.3	^*^	45 ± 5.4	^*^	30 ± 7.2	29 ± 3.9
Vitamin E as alpha-tocopherol (mg)	12	89 ± 2.2	^*^	83 ± 3.7	^*^	70 ± 3.7	60 ± 5.6
Vitamin D (D2 + D3) (μg)	10	94 ± 1.7		93 ± 2.4		92 ± 2.9	91 ± 2.5
Vitamin C (mg)	75	54 ± 3.9		52 ± 5.3		54 ± 5.0	42 ± 5.3
Thiamin (Vitamin B1) (mg)	1	11 ± 3.2	^*^	4 ± 1.4		< 3	< 3
Riboflavin (Vitamin B2) (mg)	1.1	6 ± 1.7	^*^	< 3	^*^	< 3	< 3
Niacin (mg)	12	< 3		< 3		< 3	< 3
Vitamin B6 (mg)	1.4	18 ± 4.0	^*^	8 ± 2.4		5 ± 2.0	< 3
Folate, DFE (μg)	320	17 ± 4.1	^*^	8 ± 2.2		4 ± 1.2	< 3
Vitamin B12 (μg)	2	6 ± 2.1	^*^	< 3		< 3	< 3
Calcium (mg)	800^a^	46 ± 2.9	^*^	39 ± 5.2		28 ± 2.5	24 ± 4.7
Iron (mg)	6	< 3		< 3		< 3	< 3
Magnesium (mg)	350	77 ± 2.6	^*^	68 ± 3.6	*	56 ± 4.7	46 ± 5.5
Phosphorus (mg)	580	< 3		< 3		< 3	< 3
Zinc (mg)	9.4	38 ± 4.6	^*^	24 ± 5.6		14 ± 3.8	13 ± 4.2
Copper (mg)	0.7	4 ± 1.9		< 3		< 3	< 3
Selenium (μg)	45	< 3		< 3		< 3	< 3
	**AI**	**% > AI**^‡^					
Vitamin K (μg)	120	27 ± 3.3		35 ± 3.8	^*^	42 ± 5.8	60 ± 9.5
Total choline (mg)	550	< 3		< 3		7 ± 3.1	7 ± 2.2
Potassium (mg)	3400	18 ± 2.6		27 ± 4.4	^*^	38 ± 6.7	46 ± 4.8
Dietary fiber (g)	30	5 ± 1.5		6 ± 2.1	^*^	9 ± 2.7	16 ± 3.5

^†^Model 2: covariates for day of the week (weekday/weekend), sequence of the dietary recall (day 1 or 2), whether the participant reported use of any dietary supplement in the past 30 days, and age (60–69 y, 70–79 y, 80 + y), education, race/ethnicity, marital status, PIR, comorbidities (CHD, stroke, hypertension, diabetes, cancer), kidney function (GFR and albuminuria), BMI, smoking status, physical activity, and alcohol intake

^‡^Estimated values of % < EAR or % > AI less than 3% or greater than 97% are expressed “ < 3” and “ > 97”, respectively, and the SE’s are not displayed, in accordance with the USDA’s practice in reporting nutrient adequacy. Statistical comparisons were conducted using the estimated values < 3% or > 97%

^*^Statistically significant difference (*p* < 0.05) compared to Q4

^a^EAR for males 51—70 y is 800 mg and the EAR for males 71 + y is 1000 mg. An EAR of 800 mg was selected to conservatively estimate the percent below the EAR

### Associations between nutrient intake from food plus supplements and cognitive function

When intake from food plus supplements was considered, the nutrients that showed consistent associations with cognitive function across intake analyses for both food and food plus supplements in males and females included folate, magnesium, potassium, vitamin K, and lutein and zeaxanthin (Supplementary Tables S4-S7). These associations were positive with increasing intake associated with higher CFT composite z-scores.

### Associations between nutrient intake from food and depression

Intake of vitamin A, vitamin D, vitamin C, magnesium, phosphorus, selenium, vitamin K, choline, potassium, dietary fiber, lutein + zeaxanthin, EPA, and DHA from food alone was significantly lower among females with PHQ scores ≥ 10 compared with females with a PHQ score < 10 (*p* < 0.05 for all, Table 6). The percentage of individuals consuming below the EAR for vitamin A, vitamin C, and magnesium from food alone was significantly higher among females with PHQ scores ≥ 10 compared with females with a PHQ score < 10 (*p* < 0.05 for all, Table 6). The percentage of individuals consuming above the AI was significantly lower for vitamin K, potassium, and dietary fiber from food alone among females with PHQ scores ≥ 10 compared with females with a PHQ score < 10 (*p* < 0.05 for all).

**Table 6:** Nutrient intake and adequacy from food alone by depression status among females 60^ +^ years of age^†^

		**Nutrient Intakes**			**% < EAR**^‡^		
	**PHQ Score**	** < 10**		**10 + **	** < 10**		**10 + **
		***n* = 1109**		***n* = 129**	***n* = 1109**		***n* = 129**
**Nutrient**	**EAR**	**Mean ± SE**		**Mean ± SE**	**% ± SE**		**% ± SE**
Vitamin A, RAE (μg)	500	631 ± 18	^*^	501 ± 45	34 ± 2.7	^*^	55 ± 8.7
Vitamin E as alpha-tocopherol (mg)	12	7.9 ± 0.2		7 ± 0.6	89 ± 2.1		93 ± 3.1
Vitamin D (D2 + D3) (μg)	10	4.4 ± 0.1	^*^	3.8 ± 0.3	96 ± 1.0		98 ± 0.8
Vitamin C (mg)	60	82 ± 3	^*^	64 ± 7	38 ± 2.4	^*^	55 ± 6.4
Thiamin (Vitamin B1) (mg)	0.9	1.34 ± 0.03		1.3 ± 0.07	11 ± 1.9		14 ± 3.9
Riboflavin (Vitamin B2) (mg)	0.9	1.82 ± 0.03		1.77 ± 0.09	3 ± 0.7		4 ± 1.4
Niacin (mg)	11	19.3 ± 0.4		18.2 ± 0.9	< 3		4 ± 2.1
Vitamin B6 (mg)	1.3	1.67 ± 0.03		1.52 ± 0.12	26 ± 2.1		36 ± 8.1
Folate, DFE (μg)	320	455 ± 11		447 ± 29	21 ± 2.6		23 ± 5.2
Vitamin B12 (μg)	2	4.03 ± 0.12		3.94 ± 0.34	7 ± 1.5		7 ± 3.3
Calcium (mg)	1000	830 ± 23		769 ± 44	75 ± 3.0		83 ± 4.8
Iron (mg)	5	12.4 ± 0.3		12.6 ± 0.7	< 3		< 3
Magnesium (mg)	265	266 ± 5	^*^	241 ± 12	53 ± 2.5	^*^	66 ± 6.0
Phosphorus (mg)	580	1134 ± 22	^*^	1046 ± 44	< 3		4 ± 1.3
Zinc (mg)	6.8	9 ± 0.2		8.5 ± 0.5	22 ± 2.8		29 ± 6.6
Copper (mg)	0.7	1.1 ± 0.03		1 ± 0.07	10 ± 1.3		18 ± 5.0
Selenium (μg)	45	91 ± 2	^*^	81 ± 3	< 3		3 ± 1.4
	**AI**				% >AI^‡^		
Vitamin K (μg)	120	126 ± 5	^*^	97 ± 11	63 ± 2.9	^*^	45 ± 8.0
Total choline (mg)	550	274 ± 6	^*^	242 ± 12	< 3		< 3
Potassium (mg)	3400	2415 ± 45	^*^	2130 ± 109	37 ± 2.6	^*^	22 ± 5.6
Dietary fiber (g)	30	16 ± 0.4	^*^	13.5 ± 0.9	19 ± 2.0	^*^	9 ± 3.4
Lutein + zeaxanthin (μg)	NA	1876 ± 131	^*^	1075 ± 158			
PFA 20:5 (Eicosapentaenoic) (g)	NA	0.02 ± 0.002	^*^	0.01 ± 0.002			
PFA 22:6 (Docosahexaenoic) (g)	NA	0.09 ± 0.007	^*^	0.05 ± 0.011			

^†^Model 2: covariates for day of the week (weekday/weekend), sequence of the dietary recall (day 1 or 2), whether the participant reported use of any dietary supplement in the past 30 days, and age (60–69 y, 70–79 y, 80 + y), education, race/ethnicity, marital status, PIR, comorbidities (CHD, stroke, hypertension, diabetes, cancer), kidney function (GFR and albuminuria), BMI, smoking status, physical activity, and alcohol intake

^‡^Estimated values of % < EAR or % > AI less than 3% or greater than 97% are expressed “ < 3” and “ > 97”, respectively, and the SE’s are not displayed, in accordance with the USDA’s practice in reporting nutrient adequacy. Statistical comparisons were conducted using the estimated values < 3% or > 97%

^*^Statistically significant difference (*p* < 0.05) in UI, % < EAR, or % > AI from food alone between PHQ scores of < 10 and 10 +

No significant associations were observed between nutrient intake or adequacy and PHQ scores for food alone in the male cohort (Table 7).

**Table 7:** Nutrient intake and adequacy from food alone by depression status among males 60 ^+^ years of age^†^

		**Nutrient Intakes**		**% < EAR**^‡^	
	**PHQ Score**	** < 10**	**10 + **	** < 10**	**10 + **
		***n* = 1134**	***n* = 81**	***n* = 1134**	***n* = 81**
**Nutrient**	**EAR**	**Mean ± SE**	**Mean ± SE**	**% ± SE**	**% ± SE**
Vitamin A, RAE (μg)	625	788 ± 53	626 ± 90	39 ± 3.0	59 ± 11.9
Vitamin E as alpha-tocopherol (mg)	12	9.8 ± 0.3	8.9 ± 1.1	75 ± 2.5	81 ± 8.0
Vitamin D (D2 + D3) (μg)	10	5.5 ± 0.2	4.4 ± 0.8	93 ± 1.9	97 ± 2.7
Vitamin C (mg)	75	92 ± 4	74 ± 12	50 ± 2.7	62 ± 9.1
Thiamin (Vitamin B1) (mg)	1	1.77 ± 0.03	1.76 ± 0.17	5 ± 1.1	6 ± 4.3
Riboflavin (Vitamin B2) (mg)	1.1	2.42 ± 0.07	2.4 ± 0.28	< 3	< 3
Niacin (mg)	12	27.2 ± 0.4	27.4 ± 2.4	< 3	< 3
Vitamin B6 (mg)	1.4	2.28 ± 0.05	2.18 ± 0.25	9 ± 1.8	12 ± 7.1
Folate, DFE (μg)	320	590 ± 16	560 ± 66	8 ± 1.7	12 ± 7.1
Vitamin B12 (μg)	2	5.9 ± 0.3	5.2 ± 0.9	< 3	5 ± 4.0
Calcium (mg)	800^a^	986 ± 22	997 ± 116	34 ± 1.6	33 ± 11.8
Iron (mg)	6	16.9 ± 0.3	16.4 ± 1.7	< 3	< 3
Magnesium (mg)	350	332 ± 6	323 ± 36	61 ± 1.9	65 ± 11.7
Phosphorus (mg)	580	1467 ± 18	1440 ± 123	< 3	< 3
Zinc (mg)	9.4	12.2 ± 0.2	12 ± 0.9	22 ± 3.3	25 ± 8.7
Copper (mg)	0.7	1.4 ± 0.04	1.3 ± 0.13	< 3	4 ± 3.3
Selenium (μg)	45	119 ± 1.4	120 ± 7	< 3	< 3
	**AI**			% >AI	
Vitamin K (μg)	120	125 ± 6	116 ± 26	42 ± 3.6	37 ± 14.4
Total choline (mg)	550	374 ± 6	355 ± 29	5 ± 1.5	3 ± 2.6
Potassium (mg)	3400	3067 ± 54	2888 ± 273	33 ± 2.5	26 ± 9.8
Dietary fiber (g)	30	19.5 ± 0.5	18.6 ± 2.5	9 ± 1.9	7 ± 5.3
Lutein + zeaxanthin (μg)	NA	1660 ± 125	1167 ± 343		
PFA 20:5 (Eicosapentaenoic) (g)	NA	0.03 ± 0.003	0.02 ± 0.004		
PFA 22:6 (Docosahexaenoic) (g)	NA	0.10 ± 0.010	0.08 ± 0.014		

^†^Model 2: covariates for day of the week (weekday/weekend), sequence of the dietary recall (day 1 or 2), whether the participant reported use of any dietary supplement in the past 30 days, and age (60–69 y, 70–79 y, 80 + y), education, race/ethnicity, marital status, PIR, comorbidities (CHD, stroke, hypertension, diabetes, cancer), kidney function (GFR and albuminuria), BMI, smoking status, physical activity, and alcohol intake

^a^EAR for males 51—70 y is 800 mg and the EAR for males 71 + y is 1000 mg. An EAR of 800 mg was selected to conservatively estimate the percent below the EAR

^‡^Estimated values of % < EAR or % > AI less than 3% or greater than 97% are expressed “ < 3” and “ > 97”, respectively, and the SE’s are not displayed, in accordance with the USDA’s practice in reporting nutrient adequacy. Statistical comparisons were conducted using the estimated values < 3% or > 97%

^*^Statistically significant difference (*p* < 0.05) in UI, % < EAR, or % > AI from food alone between PHQ scores of < 10 and 10 +

### Associations between nutrient intake from food plus supplements and depression

When intake from food plus supplements was considered, the nutrients that showed consistent inverse associations with depression across intake analyses for both food and food plus supplements in females included magnesium, potassium, vitamin K, dietary fiber, lutein + zeaxanthin, EPA, and DHA (Table 6 and Table S8). In males, significant inverse associations between nutrient intake and depression were only observed for food plus supplements, with vitamin A and vitamin E the only nutrients reaching significance in both intake and adequacy analyses (Table 7 and Table S9). These were inverse associations with increasing intake associated with lower depression.

## Discussion

Overall, this study shows that higher intake and adequacy of a number of different nutrients is associated with higher cognitive function in both males and females. The nutrients that showed the most consistent associations across intake and adequacy analyses from food alone in both males and females were vitamin A, vitamin E, thiamin, riboflavin, vitamin B6, folate, magnesium, potassium, zinc, vitamin K, and lutein and zeaxanthin. These associations were positive with increasing intake associated with higher CFT composite z-scores. The large number of nutrients with significant associations seems to suggest that overall nutrition and diet quality may play an important role in supporting cognition and mood. This is line with previous studies have observed that better diet quality is associated with improved cognitive performance in older adults, while conversely nutritional deficiencies can worsen cognitive deterioration [9–11]. Numerous studies have also examined the impact of single nutrients on cognitive function as discussed below.

Many of the nutrients identified are thought to support brain health through various mechanisms. Retinoic acid, the main metabolite of vitamin A, is essential for regulating synaptic plasticity in the hippocampus, a key region of the brain involved in learning and memory [30]. While animal studies have found that vitamin A deficiency leads to cognitive deficits similar to those seen in aging, the data from humans is less clear [30]. Low circulating levels of retinol potentially predict increased risk of cognitive decline, but vitamin A supplementation has not been shown to have any major effects on enhancing cognitive performance [30]. Vitamin E is one of the major antioxidants in the body, and in animal studies vitamin E deficiency leads to cognitive impairment and greater lipid peroxidation in the brain [31]. Studies in humans are limited, but one longitudinal study found that higher intakes of vitamin E were associated with reduced rates of cognitive decline in older adults [32].

We found several associations between lower B vitamin intake and lower cognitive function scores. Several B vitamins are thought to play a role in brain health and cognitive function with some more well characterized than others. Thiamin is a coenzyme in the pentose phosphate pathway which is required for the synthesis of a number of biomolecules essential for brain function [15]. However, little is known about whether thiamin status directly impacts cognitive function in healthy individuals. Similarly, riboflavin-derived coenzymes are required for most cellular enzymatic reactions, but it is unknown whether riboflavin status affects cognitive function [15]. Vitamin B6 is a necessary cofactor for numerous enzymatic reactions in the body including the synthesis of several neurotransmitters including serotonin, dopamine, norepinephrine, and gamma-aminobutyric acid. Low levels of B6 have been linked with cognitive decline [33, 34], but supplementation has not been shown to impact cognitive function in studies with an intervention period of 5–12 weeks, which may be too short of a duration for effects to be observed [35, 36]. Folate and choline are well known to play important roles in fetal brain development and brain health [15, 16]. Our results are in agreement with several cross-sectional and longitudinal studies that showed a positive association between folate intake and cognitive function [37–39]. However, randomized controlled trials (RCTs) of folate supplementation for cognition have generally shown null results [40]. This discrepancy may be influenced by the limited duration of the RCTs performed, as the effects of nutrients may occur gradually over a longer time period. Limited evidence also suggests a potential relationship between choline and cognitive performance in adults [17, 41, 42]. A recent RCT found that choline supplementation improved memory performance in older adults with age-associated memory impairment [17].

Magnesium and potassium are minerals with critical roles in basic cell function, nervous system signaling, and muscle contraction. Magnesium has an essential role in nerve transmission and neuromuscular conduction, and similar to our results, a previous analysis of NHANES data also found a positive association between magnesium intake and cognitive function [43]. This study also found that the odds of cognitive impairment was significantly reduced with increasing intake of total magnesium. We observed a positive association between potassium intake and cognitive function. Potassium is a key part of sodium–potassium exchange across cell membranes, which is vital for generating the electric potential for transmission of nerve impulses. At this time, little is known about the potential role of potassium in cognition. One case–control study found that increased serum potassium levels were associated with increased risk of mild cognitive impairment in Mexican Americans, which was unexpected and may be related to the specific population or a result of confounding caused by unadjusted factors [44], while a prospective study of community-dwelling older adults in the United States found no association between dietary potassium intake and cognitive decline [45].

Other nutrients for which we observed significant positive associations between intake and cognitive function were zinc, vitamin K, and lutein and zeaxanthin. In animal studies, zinc deficiency has been linked with defects in brain development and poor cognition. However, the evidence in humans is less clear with inconsistent effects seen in observational studies and a null effect of zinc supplementation in RCTs [46]. The authors of a systematic review and meta-analysis of zinc and cognitive function note this may be due to the methodological challenges of assessing long-term cognition effects, as well as the study populations [46]. Vitamin K accumulates in the brain and modulates the activities of key enzymes in the sphingolipid biosynthetic pathway [47]. Additionally, certain vitamin K-dependent proteins are now known to have important functions in the nervous system [47]. Our findings complement previous observational studies which have shown a correlation between low vitamin K dietary intake or serum concentration and deteriorated cognitive and behavioral performances [48]. Lutein and zeaxanthin are carotenoids that accumulate in the brain and eyes. Our results are in agreement with previous observational studies which have shown a positive correlation between lutein intake and a wide range of cognitive measures as well as lutein and zeaxanthin intervention trials showing cognitive benefits [49–52]. A prior analysis of NHANES data also found that higher dietary intake of lutein and zeaxanthin was associated with higher scores on cognitive function tests [53].

Given the large number of nutrients in this study where higher intakes were associated with better cognitive function, one might wonder whether vitamin or mineral supplementation could impact cognition. Thus far, with the exceptions of choline, lutein and zeaxanthin, the results of RCTs examining the impact of individual nutrients or multivitamins on cognition have not been particularly strong [54]. However, the effects of nutrients may occur gradually over a period of time that is likely longer than the typical duration of an RCT. Only a handful of studies have investigated long-term effects of multivitamin supplementation in large cohorts. The Physicians’ Health Study investigated the effects of multivitamin supplementation in about 6000 male physicians aged ≥ 65 years and found no effect on cognitive performance with an average follow-up of 8.5 years [55]. However, there was no baseline cognitive assessment, and the first cognitive test was performed an average of 2.5 years after randomization so shorter-term effects would be missed. More recently, the COSMOS-Mind study found that 3 years of multivitamin supplementation in about 2200 older adults (≥ 65 years of age, both men and women) led to improvements in global cognitive function, episodic memory, and executive function [56]. Improvements from baseline were seen in the first two years, and then remained stable between years 2 and 3. The authors concluded that daily use of a multivitamin supplement has the potential to improve or protect cognitive function for older adults [56].

Our exploratory analysis of nutrient intake and depression yielded results that differed by gender. In females, the nutrients that showed inverse associations with depression across both intake and adequacy analyses from food alone were vitamin A, vitamin C, magnesium, vitamin K, potassium, and dietary fiber. Similar inverse associations with magnesium, vitamin K, potassium, and dietary fiber were seen when analyses were conducted with intake from food plus supplements (Supplementary Table S8). EPA, DHA, and lutein + zeaxanthin also had inverse associations with depression across intake analyses for both food alone and food plus supplements among females. This complements a previous analysis of NHANES data which found that higher serum omega-3 fatty acids were associated with lower risk of moderately severe to severe depression [57]. Both vitamin A and vitamin C intake and adequacy were lower in women with moderate to severe depression (PHQ ≥ 10). Retinoic acid, the active metabolite of vitamin A is known to modulate neurogenesis, neuronal survival and synaptic plasticity, but no research to date has linked it with mood [14]. Some evidence suggests that vitamin C deficiency is linked with adverse mood and cognitive effects, but the nature and directionality of the association is unclear as poor diet is common in people with psychiatric disorders [58]. Magnesium intake and adequacy were lower in women with moderate to severe depression (PHQ ≥ 10). This supports previous research showing an inverse association with low magnesium intake being associated with higher rates of depression [59–62]. Similarly, vitamin K intake and adequacy were lower in women with moderate to severe depression (PHQ ≥ 10). This is in agreement with a cross-sectional study showing that people with the highest vitamin K intake had lower odds of having depressive symptoms [63]. We also found that women with moderate to severe depression (PHQ ≥ 10) had lower fiber intake and adequacy. This supports a number of previous observational studies showing that a diet high in fiber is associated with lower odds of depression [64]. Interestingly, these associations between nutrient intake and depression scores were only seen in analyses of both food alone and food plus supplements in females. No significant associations between nutrient intake and depression were observed in analyses of food alone in males, with fewer associations seen in analyses of food plus supplements in males than in females (Supplementary Table S9). This could be due in part to smaller number of males with depression included in this analysis because of lower prevalence of Moderate/Moderately Severe/Severe depression in males compared to females in this study population.

While our results show numerous associations between nutrient intake and cognitive function, our study is limited in that it can only show correlations and not causal effects. While some nutrients have well known biological roles in the brain, it is still unclear exactly how these functions might influence cognition or mood and more research would be needed to understand the mechanisms underlying these observations. Our analyses of nutrient intake and depression were limited in that only a small portion of the NHANES study population had moderate to severe depression. Additionally, calcium is the only nutrient included in this analysis that has a different EAR by age among males (800 mg for males 51 – 70 y versus 1000 mg for males > 70 y). Given the interest in determining nutrient adequacy for the study population (and not by DRI age group), the minimum EAR (800 mg) was used to conservatively estimate nutrient adequacy of calcium among males.

In summary, higher intake of nutrients from food sources was associated with higher cognitive function in males and females and with lower depression in females. Nutrient adequacy was also important as the lowest CFT composite z-score quartile had a higher percentage of individuals with shortfalls in a number of nutrients compared with the highest CFT composite z-score quartile. The large number of nutrients with significant associations seems to suggest that overall nutrition and diet quality may play an important role in supporting cognition and mood, but diet quality was not directly assessed in this study. Our findings suggest that older adults with sufficient intakes of certain essential nutrients have higher cognitive function. These results provide additional rationale for meeting sufficient nutrient intake levels per the Dietary Reference Intakes for the US population. Future studies are needed to confirm whether a well-balanced diet and/or dietary supplements which emphasize these nutrients are effective for prevention of age-related declines in cognitive function and mood.

### Supplementary Information

**Additional file 1:** **Table S1.** Comorbidities of the study population. **Table S2.** Usual nutrient intake and nutrient adequacy for foods alone and foods + supplements among males 60^+^ years of age. **Table S3.** Usual nutrient intake and nutrient adequacy for foods alone and foods + supplements among females 60^+^ years of age. **Table S4.** Nutrient intake from food + supplements by cognitive function status among females 60^+^ years of age. **Table S5.** Nutrient adequacy from food + supplements by cognitive function status among females 60^+^ years of age. **Table S6.** Nutrient intake from food + supplements by cognitive function status among males 60^+^ years of age. **Table S7.** Nutrient adequacy from food + supplements by cognitive function status among males 60^+^ years of age. **Table S8.** Nutrient intake and adequacy from food + supplements by depression status among females 60^+^ years of age. **Table S9.** Nutrient intake and adequacy from food + supplements by depression status among males 60^+^ years of age.

## Data Availability

The National Health and Nutrition Examination Survey (NHANES) data is freely available online and can be accessed at the following url: https://www.cdc.gov/nchs/nhanes/index.htm.

## Abbreviations

AI: Adequate intake
ACR: Albumin-to-creatine ratio
BMI: Body mass index
CERAD: Consortium to Establish a Registry for Alzheimer’s Disease
CFT: Cognitive function tests
DHA: Docosahexaenoic acid
DRI: Dietary reference intakes
DSST: Digit Symbol Substitution Test
EAR: Estimated average requirement
EPA: Eicosapentaenoic acid
GFR: Glomerular filtration rate
NASEM: National Academy of Science, Engineering, and Medicine
NCI: National Cancer Institute
NHANES: National Health and Nutrition Examination Survey
PHQ: Patient Health Questionnaire
PIR: Poverty-to-income ratio
RCT: Randomized controlled trial
SE: Standard error
UI: Usual intake
